# Supplementary material for: Binding of NUFIP2 to Roquin promotes recognition and regulation of ICOS mRNA
Source: Nat Commun. 2018 Jan 19;9:299. doi: 10.1038/s41467-017-02582-1 (PMC5775257; doi:10.1038/s41467-017-02582-1)
Supplement: Supplementary file 1 — Supplementary Information [file 41467_2017_2582_MOESM1_ESM.pdf]

Supplementary Table 1: Primers used for cloning

Gene	Primer	Sequence
NUFIP2	Fw	GTCGACCATGGAGGAGAAGCCCGGC
	Rev	GCGGCCGCTCATTGATCTGGACTATCCATGG
NUFIP2 aa 255–411	Fw	AAGTTCTGTTTCAGGGCCCGAAACAGGGACTTGAA
	Rev	GCGGCCGCTCATTGATCTGGACTATCCATGG
mFmrp aa 1–132	Fw	AAGTTCTGTTTCAGGGCCCGATGGAGGAGCTGGTG
	Rev	ATGGTCTAGAAAGCTTTACTCCAGCTTGATCTTATG
NUFIP2 aa 411–695	Fw	GTCGACCATGTCTGCCAACTTTTCTAATGGGC
	Rev	GCGGCCGCTCATTGATCTGGACTATCCATGG
ICOS Δ700–800	Fw	ATGAAGTCAGGCCTCTGGTATTTC
	Rev	GAGACTCGAGTAAGTCAGACTCTCCGTGGTCC

Supplementary Table 2: Primers used for site-directed mutagenesis

Primer	Sequence	Mutation
Fw	CTGTTAAAATCCCCATTGACTGATAGTGTCATTCTTCCAGATTGAACA AAAC	ICOS M1 M1 M3
Rev	GTTTTGTTCAATCTGGAAGAATGACACTATCAGTCAATGGGGATTTTA ACAG	ICOS M1 M1 M3
Fw	TTTACAAGTGAGACCCGATATGTCATTATGCATACTTAGAGGAGCGTA AGCATGTGTAATGCTGGATGTGTAC	ICOS M2, M1 M2, M2 M3
Rev	GTACACATCCAGCATTACACATGCTTACGCTCCTCTAAGTATGCATAA TGACATATCGGGTCTCACTTGTAAA	ICOS M2, M1 M2, M2 M3
Fw	TACTAGATTCAAATTACAAGTTCAGATCTGTACTGTACACATCCAGCA TTAC	ICOS M3, M1 M2 M3
Rev	GTAATGCTGGATGTGTACAGTACAGATCTGAACTTGTAATTTGAATCT AGTA	ICOS M3, M1 M2 M3
Fw	CTTAAGATAATATAAGTATGCATAATGGCAATGTTTCCTACCACTAAC TTGAAC	ICOS delA
Rev	GTTCAAGTTAGTGGTAGGAAACATTGCCATTATGCATACTTATATTAT CTTAAG	ICOS delA
Fw	CTGGAAGAATGACTGTATCAGTCAACCGGAATTGAAAGCAAAT	ICOS delB
Rev	ATTTGCTTTCAATTCCGGTTGACTGATACAGTCATTCTTCCAG	ICOS delB
Fw	GACTGTATCAGTCAATGGGGATTTTCCCGATATGTCATTATGC	ICOS delC
Rev	GCATAATGACATATCGGGAAAATCCCCATTGACTGATACAGTC	ICOS delC
Fw	TTGCTGCTCACACCAGGTTTACTATCAGATGACTTCCTAAGCATATCA CCCACAGCAGGTTTTC	siNUFIP2#2-res. NUFIP2
Rev	GAAAACCTGCTGTGGGTGATATGCTTAGGAAGTCATCTGATAGTAAA CCTGGTGTGAGCAGCAA	siNUFIP2#2-res. NUFIP2
Fw	TTCAAGTCCCTGTCATTTTAAGGTTGGGACACTGGTC	NUFIP2 1–255
Rev	GACCAGTGTCCCAACCTTAAAATGACAGGGACTTGAA	NUFIP2 1–255
Fw	CCTATGTCAGCGCTGAAATCTGTTACTTGATCTGCCAACTTTTC	NUFIP2 1–411

Primer	Sequence	Mutation
Rev	GAAAAGTTGGCAGATCAAGTAACAGATTTCAGCGCTGACATAGG	NUFIP2 1–411

Supplementary Table 3: Primers used as templates for *in vitro* transcription

RNA fragments	Primer sequence
**ICOS (nts 2183–2271)**	TGTCATTATGCATACTTATATTATCTTAAGCATGTGTAATGCTGG ATGTGTACAGTACAGTACTGAACTTGTAATTTAATCTAGTATG
**ICOS (nts 2193–2216)**	CATGCTTAAGATAATATAAGTATGCCCTATAGTGAGTCGTATTA
**ICOS (nts 2224–2253)**	CAAGTTCAGTACTGTACTGTACACATCCAGCCCTATAGTGAGTC GTATTA
**ICOS (nts 2251–2271)**	CATACTAGATTCAAATTACAACCCTATAGTGAGTCGTATTA
**ICOS (nts 2183–2226)**	CAGCATTACACATGCTTAAGATAATATAAGTATGCATAATGACAC CCTATAGTGAGTCGTATTA
**Ox40 (nts 1064-1126)**	GGTAGTATGCATAGCATACATAGGAAAGCCCAGAGCCAGCCCA GCACCTAGAACGGTGTGGACCCTATAGTGAGTCGTATTA

Supplementary Table 4: qPCR primers and corresponding universal probe library number

Target	Forward primer	Reverse primer	Probe
CNOT1	TTGGAGGACTTTCATCACAGC	GAGCACCAGTTCCTATACCAGTC	27
HPRT	TCCTCCTCAGACCGCTTTT	CCTGGTTCATCATCGCTAATC	95
ICOS	GGATGCATACTTATTTGTTGGCTTA	TGTATTCACCGTTAGGGTCGT	47
NUFIP2	CGCTGAAACATGAGCAGAAA	TCCAGCATTACCGTTTAGTTCA	67
STAU1	ATGGTATCGGCAAGGATGTG	TGGTCCAACTCAGACAGCAA	46
YWHAZ	CGCTAATAATGCAGTTACTGAGAGA	TTGGAAGGCCGGTTAATTTT	2
RC3H1	TGGACAACCAGAACCACAAA	GATCCATTTGGTACATCACTGCT	73
RC3H2	AAGGTTGGCGCTAATGGTC	CAGGAGTCTTGGGTGGAGAA	3

Supplementary Table 5: Sequences used for sequence and structure conservation analysis

Species	Full species name	Gi number	Accession number
B. b. bison	*Bison bison bison*	742207131	XM 010861022.1
B. bubalis	*Bubalus bubalis*	594062074	XM 006055039.1
B. mutus	*Bos mutus*	555984917	XM 005904279.1
B. taurus	*Bos taurus*	77735504	NM 001034275.1
C. circus	*Capra circus*	548454576	XM 005676405.1
C. jacchus	*Callithrix jacchus*	675665233	XM 008999272.1
C. lanigera	*Chinchilla lanigera*	533166791	XM 005397010.1
C. sabaeus	*Chlorocebus sabaeus*	635060004	XM 007965946.1
D. novemcinctus	*Dasypus novemcinctus*	488523590	XM 004453622.1
E. caballus	*Equus caballus*	545191678	XM 005601696.1
G. g. gorilla	*Gorilla gorilla gorilla*	426338330	XM 004033087.1

H. sapiens	*Homo sapiens*	251823951	NM 012092.3
M. domestica	*Monodelphis domestica*	612047130	XM 007501882.1
M. fascicularis	*Macaca fascicularis*	544477052	XM 005574018.1
M. mulatta	*Macaca mulatta*	388454902	NM 001266989.1
M. p. furo	*Mustela putorius furo*	511889060	XM 004764206.1
O. aries	*Ovis aries*	426221393	XM 004004846.1
O. cuniculus	*Oryctolagus cuniculus*	655828861	XM 008259011.1
P. anubis	*Papio anubis*	685578022	XM 003907838.2
P. hodgsonii	*Pantholops hodgsonii*	556770177	XM 005980222.1
P. paniscus	*Pan paniscus*	397500230	XM 003820780.1
R. roxellana	*Rhinopithecus roxellana*	724947902	XM 010352637.1
S. b. boliviensis	*Saimiri boliviensis boliviensis*	403267073	XM 003925628.1
S. scrofa	*Sus scrofa*	113205633	NM 001044546.1

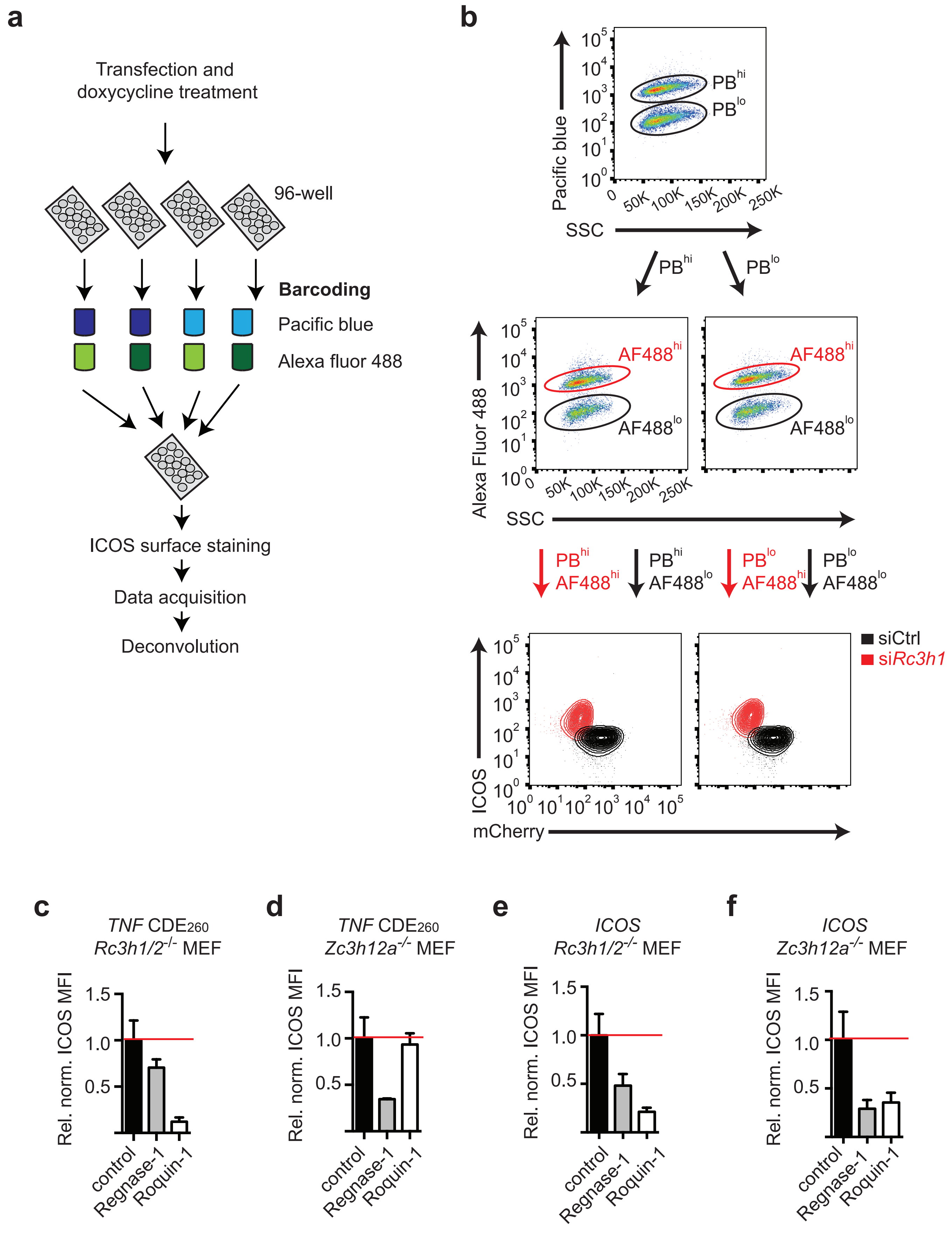
a
Transfection and doxycycline treatment
96-well
Barcoding
Pacific blue
Alexa fluor 488
ICOS surface staining
Data acquisition
Deconvolution
b
Pacific blue
SSC
PBhi
PBlo
Alexa Fluor 488
AF488hi
AF488lo
PBhi AF488hi
PBhi AF488lo
PBlo AF488hi
PBlo AF488lo
siCtrl
siRc3h1
ICOS
mCherry
c
TNF CDE260 Rc3h1/2-/- MEF
d
TNF CDE260 Zc3h12a-/- MEF
e
ICOS Rc3h1/2-/- MEF
f
ICOS Zc3h12a-/- MEF
Rel. norm. ICOS MFI
control
Regnase-1
Roquin-1

Supplementary Figure 1: Arrayed RNAi screening identifies Regnase-1 as a target-specific cofactor of Roquin

(**a**) Schematic representation of the transfection, barcoding and data acquisition workflow of the screen. Fluorescent cell barcoding of transfected cells allowed the pooling of four 96-well plates into one for simultaneous ICOS staining and data acquisition. (**b**) Representative deconvolution of barcoded samples. HeLa reporter cells transfected with non-targeting control siRNA (black populations) or Roquin-targeting siRNA pools (red populations) were stained according to the barcoding protocol and analyzed by flow cytometry. Samples were first deconvoluted based on their Pacific blue (PB) fluorescence intensity into high (PB^{hi}) and low (PB^{lo})-expressing cells. Second, Alexa fluor 488 high ($AF488^{hi}$) and low ($AF488^{lo}$)-expressing cells were separated, leading to the deconvolution into the four original samples. ICOS and mCherry expression was plotted for each sample and demonstrate little cross-contamination. (**c–f**) *Rc3h1/2*$^{-/-}$ or *Zc3h12a*$^{-/-}$ MEF cells were transduced with the CDE_{260} (**c,d**) or *ICOS* 3′-UTR (**e,f**) reporters that were cloned downstream of the CDS of *ICOS*. The cells were superinfected with Roquin-1– or Regnase-1–encoding retroviruses and ICOS expression was analyzed by flow cytometry after 48 h. ICOS MFI of each sample was normalized to the average of the uninfected control (red line). Error bars in **c–f** represent mean and SD of three independent experiments.

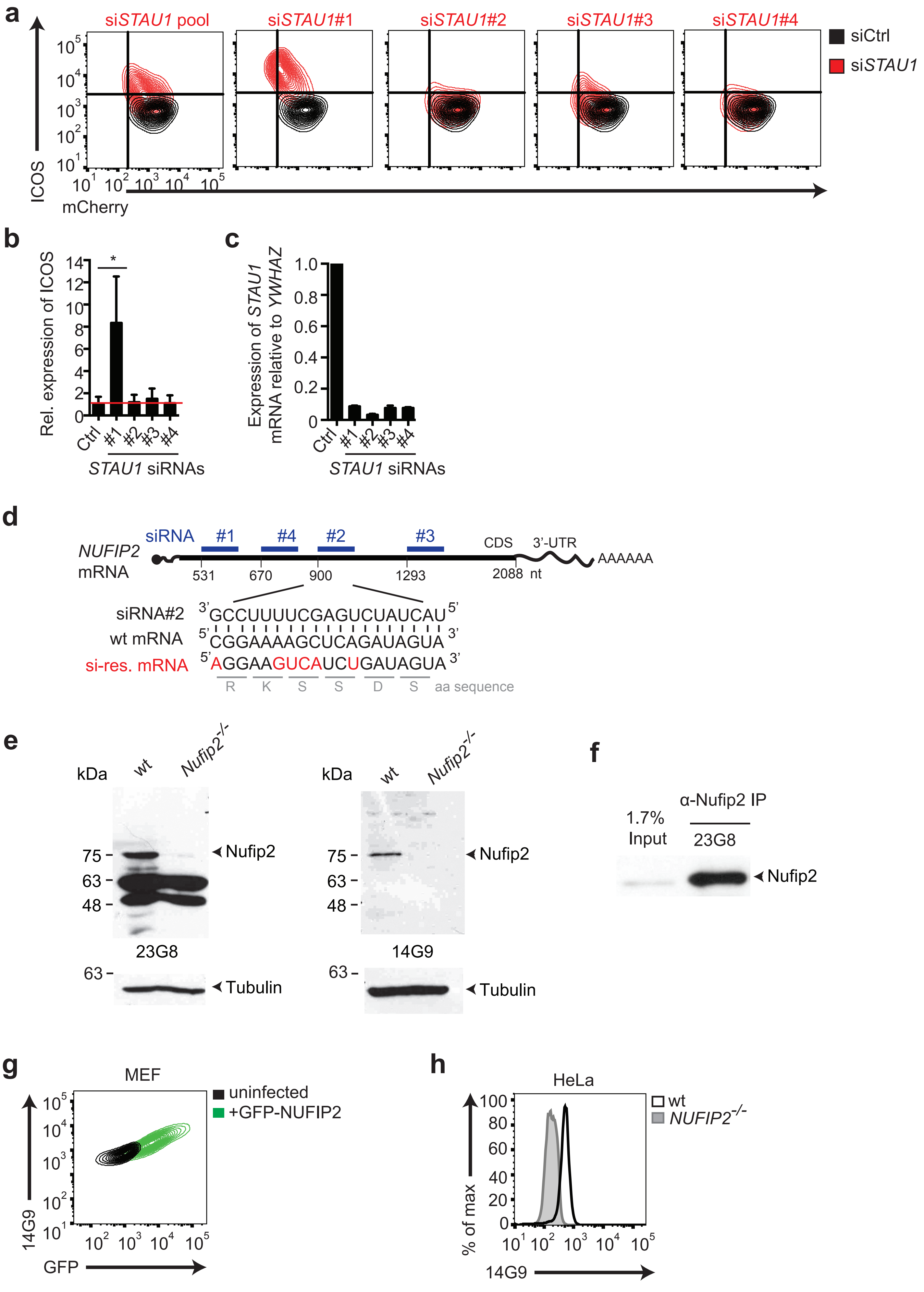
a
siSTAU1 pool
siSTAU1#1
siSTAU1#2
siSTAU1#3
siSTAU1#4
siCtrl
siSTAU1
ICOS
mCherry
b
Rel. expression of ICOS
Ctrl
#1
#2
#3
#4
STAU1 siRNAs
c
Expression of STAU1 mRNA relative to YWHAZ
Ctrl
#1
#2
#3
#4
STAU1 siRNAs
d
NUFIP2 mRNA
siRNA
#1
#4
#2
#3
CDS
3'-UTR
AAAAAA
531
670
900
1293
2088 nt
siRNA#2 3' GCCUUUUCGAGUCUAUCAU 5'
wt mRNA 5' CGGAAAAGCUCAGAUAGUA 3'
si-res. mRNA 5' AGGAAGUCAUCUGAUAGUA 3'
R K S S D S aa sequence
e
kDa
wt
Nufip2-/-
75
63
48
Nufip2
23G8
Tubulin
14G9
f
1.7% Input
α-Nufip2 IP
23G8
Nufip2
g
MEF
uninfected
+GFP-NUFIP2
14G9
GFP
h
HeLa
wt
NUFIP2-/-
% of max
14G9

Supplementary Figure 2: Deconvolution of the STAU1 siRNA pool and validation of Nufip2-specific monoclonal antibodies

(**a**) Flow cytometry analysis of ICOS and mCherry expression in HeLa reporter cells treated with individual or pooled *STAU1* siRNAs (red) or non-targeting control siRNAs (siCtrl) (black). Prior to analysis, cells were treated with doxycycline for 18 h to induce Roquin-1 overexpression. (**b**) Quantified ICOS MFI of cells shown in **a.** Expression levels were normalized to siCtrl-treated cells. (**c**) qPCR analysis of *STAU1* mRNA expression in cells from **a**. Expression was calculated relative to the reference gene *YWHAZ* and normalized to siCtrl-treated cells. (**d**) Schematic representation of individual siRNAs targeting *NUFIP2* mRNA. Mutations to generate siRNA#2-resistant NUFIP2 are shown in red. (**e**) Immunoblot analysis of Nufip2 expression in lysates from wild-type (wt) and Nufip2–deficient ($Nufip2^{-/-}$) MEF cells. Blots were probed with the newly generated monoclonal antibodies 23G8 and 14G9. (**f**) Immunoprecipitation of Nufip2 from MEF cell lysates using the monoclonal antibody 23G8. Immunoblot analysis of Nufip2 was performed with a polyclonal Nufip2 antibody. (**g**) Flow cytometry analysis of intracellular Nufip2 and GFP expression in lysates from MEF cells that were transduced retrovirally with GFP-NUFIP2 or left uninfected. Intracellular Nufip2 was stained with the 14G9 antibody. (**h**) Flow cytometry analysis of intracellular Nufip2 as in (**g**) was performed in wild-type (wt) and Nufip2–deficient HeLa cells. Error bars in **b** and **c** represent mean and SD of three independent experiments. In **a** the representative of three independent experiments are shown. Statistical significance in **b** was calculated with one-way Anova Kruskal-Wallis test followed by Dunn's multiple comparisons test (*$p<0.05$).

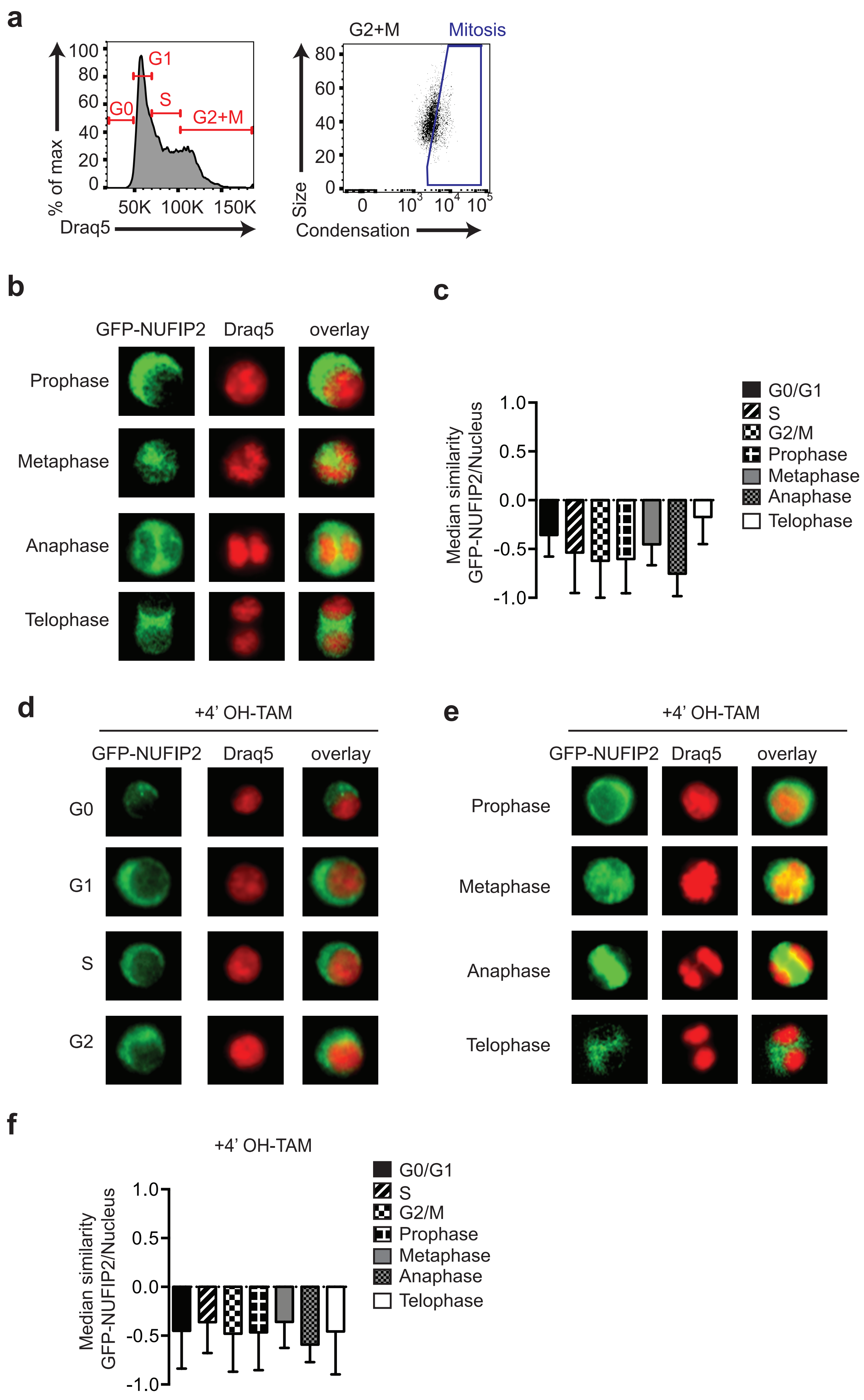
a
100
80
60
40
20
0
G1
G0
S
G2+M
% of max
50K 100K 150K
Draq5
G2+M
Mitosis
80
60
40
20
0
Size
0 10^3 10^4 10^5
Condensation
b
GFP-NUFIP2 Draq5 overlay
Prophase
Metaphase
Anaphase
Telophase
c
Median similarity GFP-NUFIP2/Nucleus
1.0
0.5
0.0
-0.5
-1.0
G0/G1
S
G2/M
Prophase
Metaphase
Anaphase
Telophase
d
+4' OH-TAM
GFP-NUFIP2 Draq5 overlay
G0
G1
S
G2
e
+4' OH-TAM
GFP-NUFIP2 Draq5 overlay
Prophase
Metaphase
Anaphase
Telophase
f
+4' OH-TAM
Median similarity GFP-NUFIP2/Nucleus
1.0
0.5
0.0
-0.5
-1.0
G0/G1
S
G2/M
Prophase
Metaphase
Anaphase
Telophase

Supplementary Figure 3: NUFIP2 shows a diffuse, cell cycle-independent cytoplasmic localization in mouse CD4$^+$ T cells

Flow cytometry imaging analysis of GFP-Nufip2 localization in CD4$^+$ T cells isolated from *Rc3h1*$^{fl/fl}$*; Rc3h2*$^{fl/fl}$*; Cd4-Cre-ERT2; rtTA* mice. Cells were activated with anti-CD3 and anti-CD28 under Th1 conditions for 48 h, transduced with doxycycline-inducible GFP-NUFIP2 and amplified in IL-2 medium for two days. Before analysis, cells were treated with doxycycline for 24 h to induce GFP-NUFIP2 expression. Localization of GFP-NUFIP2 was compared between untreated cells (**a–c**) and after *in vitro* treatment with 4′ OH-tamoxifen to induce *Rc3h1-2*$^{fl/fl}$ deletion (**d–f**). (**a**) Representative gating on cells in different phases of the cell cycle. (**b, d, e**) Flow cytometry imaging analysis of GFP-NUFIP2 localization in different cell cycle phases. (**c, f**) GFP-NUFIP2/Draq5 similarity calculations for different phases of the cell cycle. **b**, **d** and **e** show representatives of three independent experiments. In **c** and **f** error bars indicate mean and SD of three independent experiments.

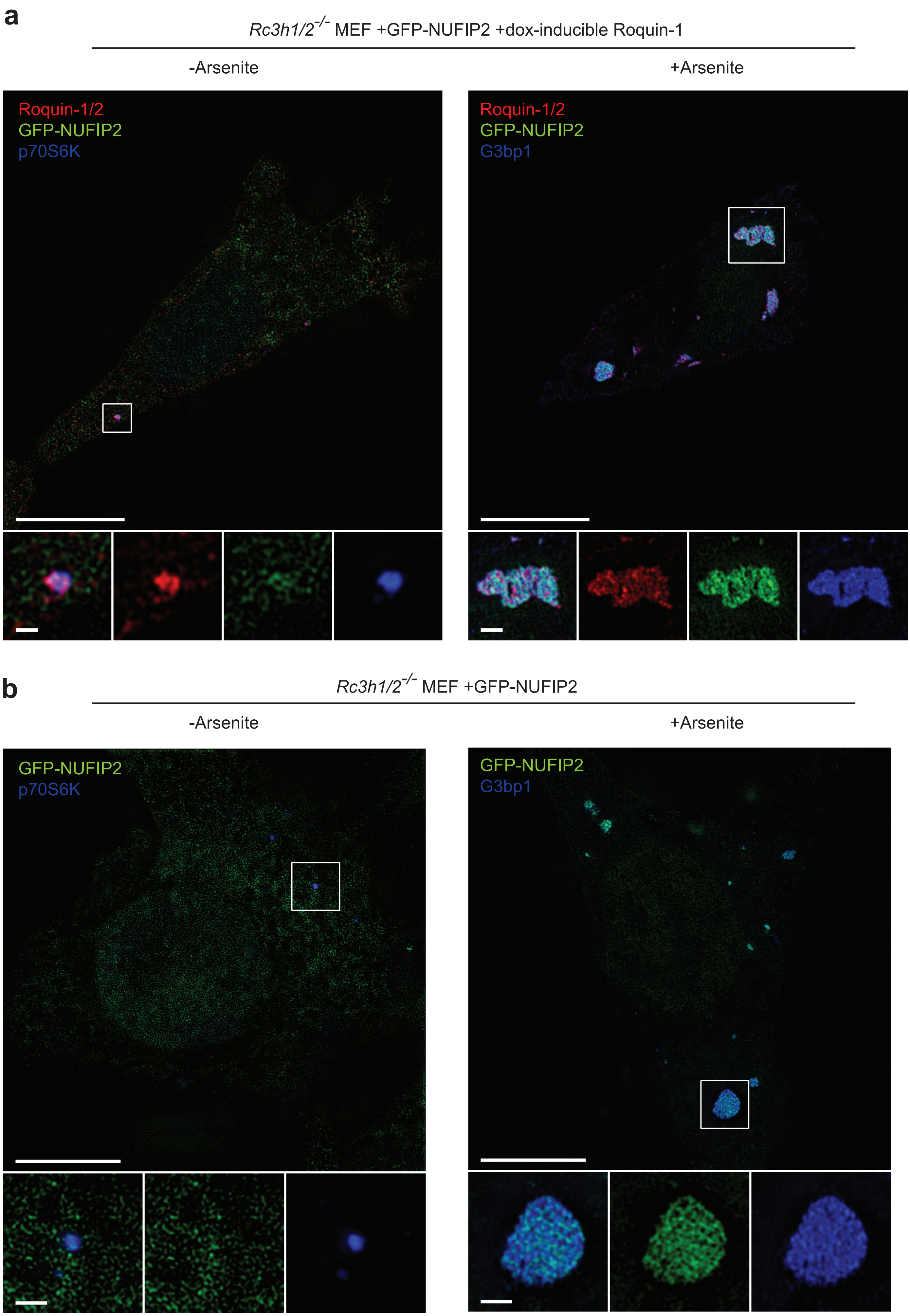
a
Rc3h1/2-/- MEF +GFP-NUFIP2 +dox-inducible Roquin-1
-Arsenite
+Arsenite
Roquin-1/2
GFP-NUFIP2
p70S6K
Roquin-1/2
GFP-NUFIP2
G3bp1
b
Rc3h1/2-/- MEF +GFP-NUFIP2
-Arsenite
+Arsenite
GFP-NUFIP2
p70S6K
GFP-NUFIP2
G3bp1

Supplementary Figure 4: Colocalization of NUFIP2 with P body markers depends on Roquin

(**a**) Super-resolution microscopy of ectopic Roquin-1 (red) and GFP-NUFIP2 (green) localization either with the P body marker Edc4 (stained in the cytoplasm with an antibody against p70S6K) (blue) in unstressed cells (-Arsenite) or with the stress granule marker G3bp1 (blue) upon induction of oxidative stress (+Arsenite). Zoom (bottom panel), enlargement of areas outlined above. Scale bars: 10 and 1 µm. (**b**) Super-resolution microscopy of GFP-NUFIP2 and Edc4 (blue) localization in unstressed cells (-Arsenite) or of GFP-NUFIP2 and G3bp1 (blue) upon induction of oxidative stress (+Arsenite) in Roquin–deficient (*Rc3h1/2*$^{-/-}$) MEF cells. Representatives of ten individually acquired cells are shown.

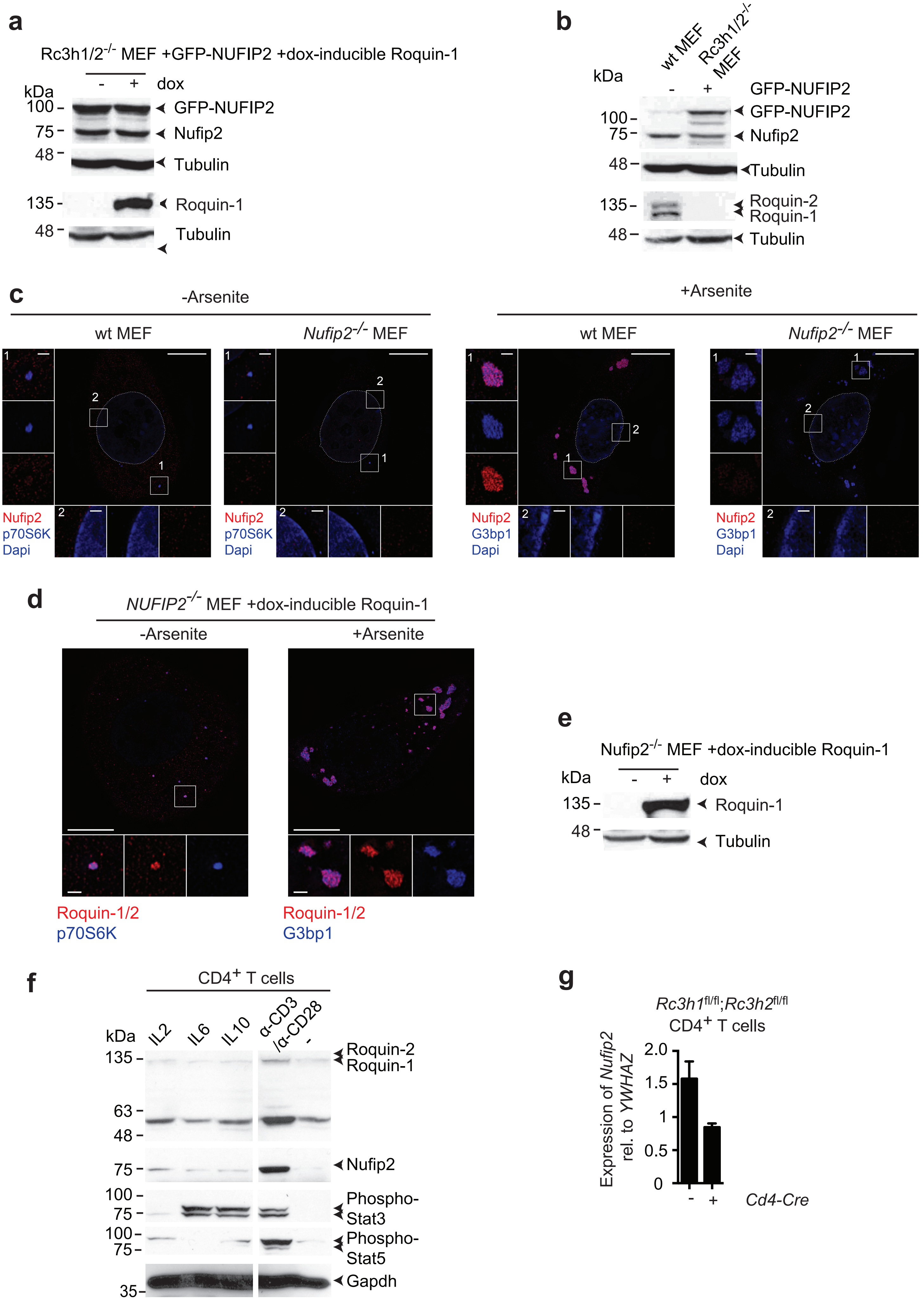

a
Rc3h1/2-/- MEF +GFP-NUFIP2 +dox-inducible Roquin-1
- + dox
kDa
100 GFP-NUFIP2
75 Nufip2
48 Tubulin
135 Roquin-1
48 Tubulin
b
wt MEF
Rc3h1/2-/- MEF
kDa
- + GFP-NUFIP2
100 GFP-NUFIP2
75 Nufip2
48 Tubulin
135 Roquin-2
Roquin-1
48 Tubulin
c
-Arsenite
+Arsenite
wt MEF
Nufip2-/- MEF
Nufip2
p70S6K
Dapi
G3bp1
d
NUFIP2-/- MEF +dox-inducible Roquin-1
-Arsenite
+Arsenite
Roquin-1/2
p70S6K
G3bp1
e
Nufip2-/- MEF +dox-inducible Roquin-1
kDa
- + dox
135 Roquin-1
48 Tubulin
f
CD4+ T cells
kDa
IL2
IL6
IL10
α-CD3 /α-CD28
-
135 Roquin-2
Roquin-1
63
48
75 Nufip2
100
75 Phospho-Stat3
100
75 Phospho-Stat5
35 Gapdh
g
Rc3h1fl/fl;Rc3h2fl/fl
CD4+ T cells
Expression of Nufip2 rel. to YWHAZ
2.0
1.5
1
0.5
0
- +
Cd4-Cre

Supplementary Figure 5: Subcellular localization of Roquin and Nufip2

(**a**) Immunoblot analysis of Nufip2 and Roquin expression in lysates from Roquin–deficient (*Rc3h1/2*$^{-/-}$) MEF cells that were transduced with doxycycline-inducible Roquin by retroviral infection and superinfected with GFP-NUFIP2. Cells were treated with doxycycline for 18 h (+dox) or left untreated (-dox). (**b**) Immunoblot analysis of Nufip2 and Roquin expression in lysates from *Rc3h1/2*$^{-/-}$ cells that were transduced with GFP-NUFIP2 by retroviral infection or left untransduced. (**c**) Super-resolution microscopy of the colocalization of endogenous Nufip2 (red) either with the P body marker Edc4 (p70S6K, blue) in unstressed cells (-Arsenite) or with the stress granule marker G3bp1 (blue) upon induction of oxidative stress (+Arsenite) in wild-type or Nufip2–deficient (*Nufip2*$^{-/-}$) MEF cells. Zoom (left and bottom panels), enlargement of areas outlined above. Nuclear membranes are indicated by white dotted lines. Scale bars: 10 and 1 µm. (**d**) Super-resolution microscopy of the colocalization of ectopic Roquin-1 (red) either with Edc4 (blue) in unstressed cells (-Arsenite) or with G3bp1 (blue) upon induction of oxidative stress (+Arsenite) in Nufip2–deficient (*Nufip2*$^{-/-}$) MEF cells with cassettes for doxycycline-inducible Roquin overexpression that were treated with doxycycline for 18 h (+dox) or left untreated (-dox). Zoom (bottom panel), enlargement of areas outlined above. Scale bars: 10 and 1 µm. The cells used in (**d**) were analyzed in (**e**) by immunoblot analysis for Roquin expression. (**f**) Immunoblot analysis of Nufip2 expression in mouse $CD4^+$ T cells upon exposure to different cytokines or anti-CD3 and anti-CD28 for 20 h. Following isolation, cells were activated with anti-CD3 and anti-CD28 under Th1 conditions for 40 h and amplified in IL-2 medium for four days before cytokine stimulation. Phospho-Stat3 and Phospho-Stat5 serve as controls for cytokine stimulation and Gapdh served as a loading control. (**g**) qPCR analysis of *Nufip2* mRNA expression in cell used in **Fig. 4e**. Expression was calculated relative to the reference gene *Ywhaz* and normalized to untransduced cells. In **c** and **d** representatives of ten individually acquired cells are shown. In **f** one representative of two independent experiments is shown. Error bars in **g** indicate mean and SD of two independent experiments.

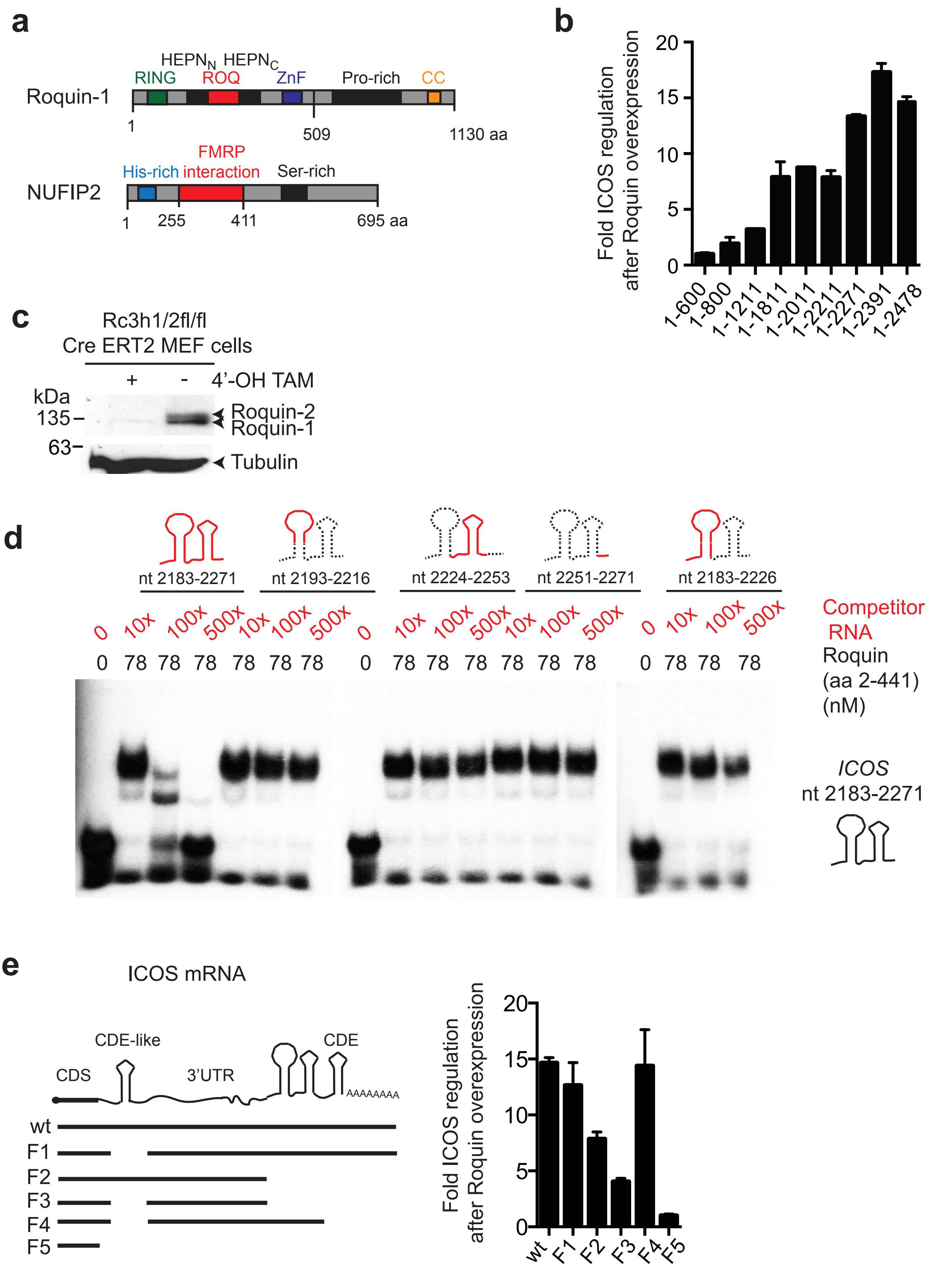
a
Roquin-1
RING
HEPN$_N$ HEPN$_C$
ROQ
ZnF
Pro-rich
CC
1
509
1130 aa
NUFIP2
His-rich
FMRP interaction
Ser-rich
1
255
411
695 aa
b
Fold ICOS regulation after Roquin overexpression
0
5
10
15
20
1-600
1-800
1-1211
1-1811
1-2011
1-2211
1-2271
1-2391
1-2478
c
Rc3h1/2fl/fl Cre ERT2 MEF cells
+
-
4'-OH TAM
kDa
135
63
Roquin-2
Roquin-1
Tubulin
d
nt 2183-2271
nt 2193-2216
nt 2224-2253
nt 2251-2271
nt 2183-2226
0 10x 100x 500x 10x 100x 500x 0 10x 100x 500x 10x 100x 500x 0 10x 100x 500x
Competitor RNA
0 78 78 78 78 78 78 0 78 78 78 78 78 78 0 78 78 78
Roquin (aa 2-441) (nM)
ICOS nt 2183-2271
e
ICOS mRNA
CDE-like
CDE
CDS
3'UTR
AAAAAAAA
wt
F1
F2
F3
F4
F5
Fold ICOS regulation after Roquin overexpression
0
5
10
15
20
wt
F1
F2
F3
F4
F5

Supplementary Figure 6: Mapping of Roquin *cis*-elements in *ICOS* mRNA

(**a**) Domain organization of mouse Roquin-1 and human NUFIP2 proteins. HEPN, higher eukaryotes and prokaryotes nucleotide-binding domain; ZnF, zinc finger; CC, coiled-coil region. (**b**) Reporter regulation of *ICOS* 3′-UTR deletion constructs in response to overexpressed Roquin-1 in *Rc3h1/2*$^{-/-}$ MEF cells with doxycycline-inducible Roquin-1-P2A-mCherry. Fold regulation was determined by dividing (ICOS MFI –dox/ICOS MFI +dox) and normalized to the regulation of ICOS 1-600. (**c**) Representative immunoblot analysis of Roquin expression in *Rc3h1*$^{fl/fl}$*; Rc3h2*$^{fl/fl}$*; Cre-ERT2* MEF cell lysates. Before lysis, cells were treated with 4' OH-TAM for 3 days or left untreated. (**d**) EMSA with 78 nM of Roquin-1 (aa 2–441) incubated with *ICOS* (nt 2183–2271) RNA and competed with unlabeled *ICOS* RNA fragments in 10 to 500-fold excess as indicated. (**e**) Reporter regulation of *ICOS* 3′-UTR deletion constructs F1-F5 (schematic representation on the left) in response to overexpressed Roquin-1 in *Rc3h1/2*$^{-/-}$ MEF cells with doxycycline-inducible Roquin-1-P2A-mCherry. Fold regulation was calculated as in (**b**). Error bars in **b** and **e** indicate mean and SD of two independent experiments. In **d** one representative of three independent experiments is shown.

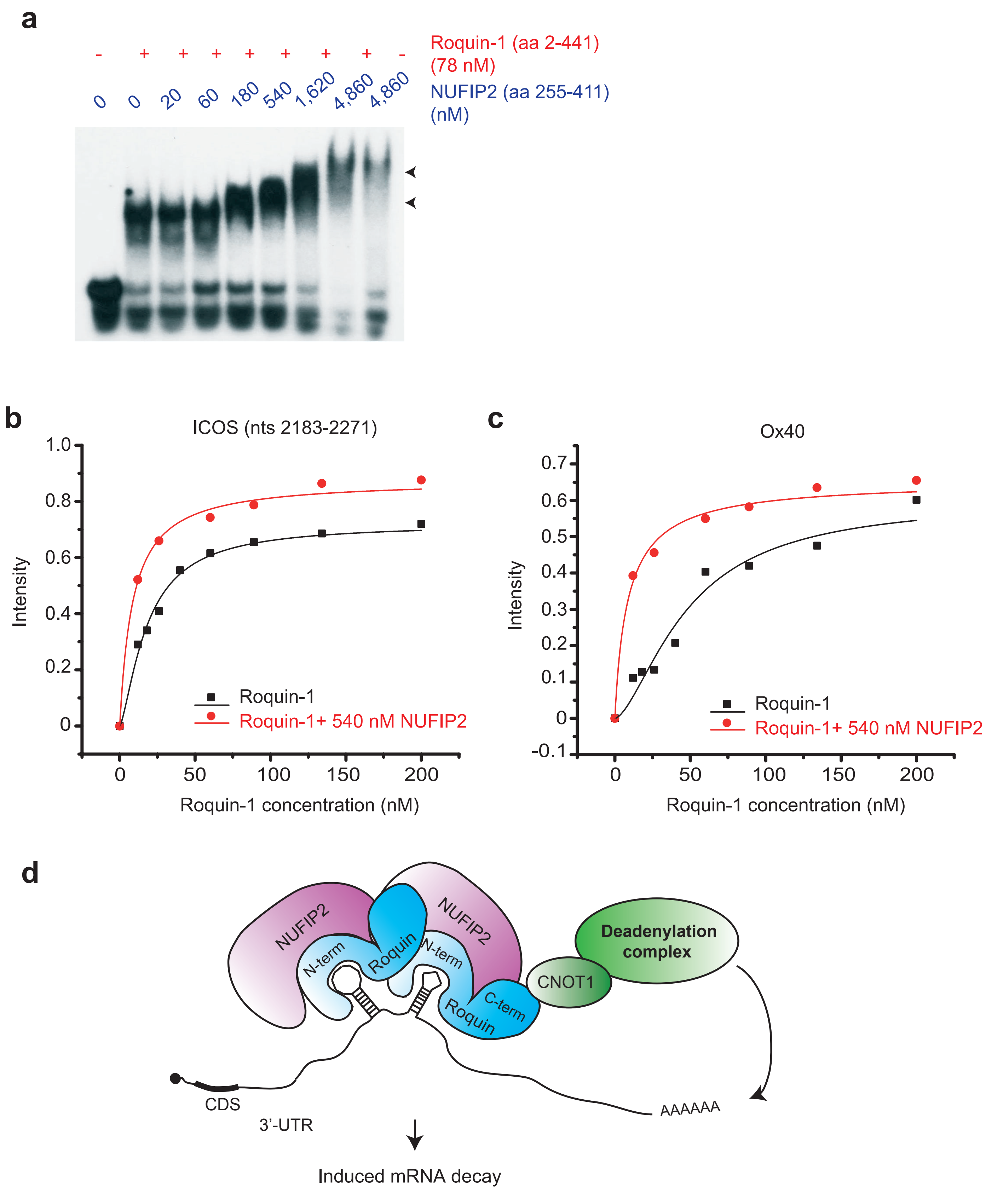

a
- + + + + + + + -
Roquin-1 (aa 2-441) (78 nM)
0 0 20 60 180 540 1,620 4,860 4,860
NUFIP2 (aa 255-411) (nM)
b
ICOS (nts 2183-2271)
Intensity
Roquin-1 concentration (nM)
Roquin-1
Roquin-1+ 540 nM NUFIP2
c
Ox40
Intensity
Roquin-1 concentration (nM)
Roquin-1
Roquin-1+ 540 nM NUFIP2
d
NUFIP2
N-term
Roquin
NUFIP2
N-term
C-term
Roquin
CNOT1
Deadenylation complex
CDS
3'-UTR
AAAAAA
Induced mRNA decay

Supplementary Figure 7: Cooperative binding of NUFIP2 and Roquin to target mRNA

(**a**) EMSA with increasing amounts (0–4,860 nM) of NUFIP2 (aa 255-411) incubated with *ICOS* (nt 2183–2271) RNA in the presence or absence of 78 nM Roquin-1 (aa 2–441). (**b**) Representative binding curves from three independent EMSA experiments with *ICOS* (nt 2183–2271) RNA and increasing amounts (0-200 nM) of Roquin-1 in presence or absence of 540 nM NUFIP2. Arrowheads indicate NUFIP2-induced supershifts. (**c**) Representative binding curves as in (**b**) but with Ox40 (nt 1064-1126) RNA instead. (**d**) Model of Roquin-mediated post-transcriptional repression of *ICOS* mRNA involving NUFIP2 as a cofactor. Full repression of *ICOS* and *Ox40* mRNA requires Roquin binding to a tandem stem-loop. NUFIP2 contributes to Roquin-mediated *ICOS* repression by cooperative binding of the *cis*-element. This may either involve a change in Roquin folding to increase interaction with specific target sites, or through the simultaneous interaction with Roquin and NUFIP2 the *ICOS* mRNA is bound with increased avidity. Interactions of Roquin with CNOT1 promote target mRNA deadenylation and mRNA decay.

Figure 1a

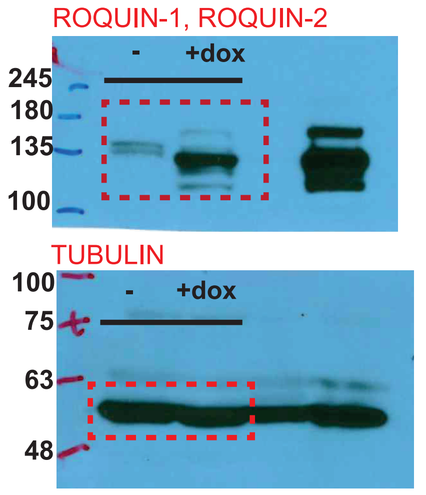

Figure 3c (Cnot1)

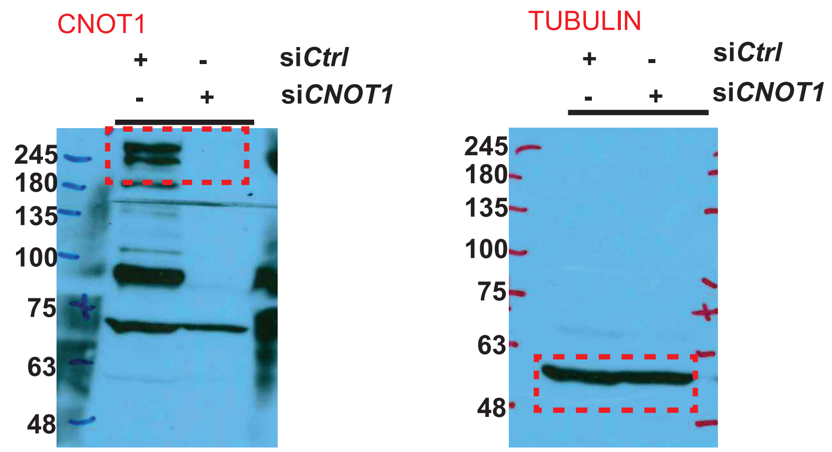

Figure 3c (Nufip2)

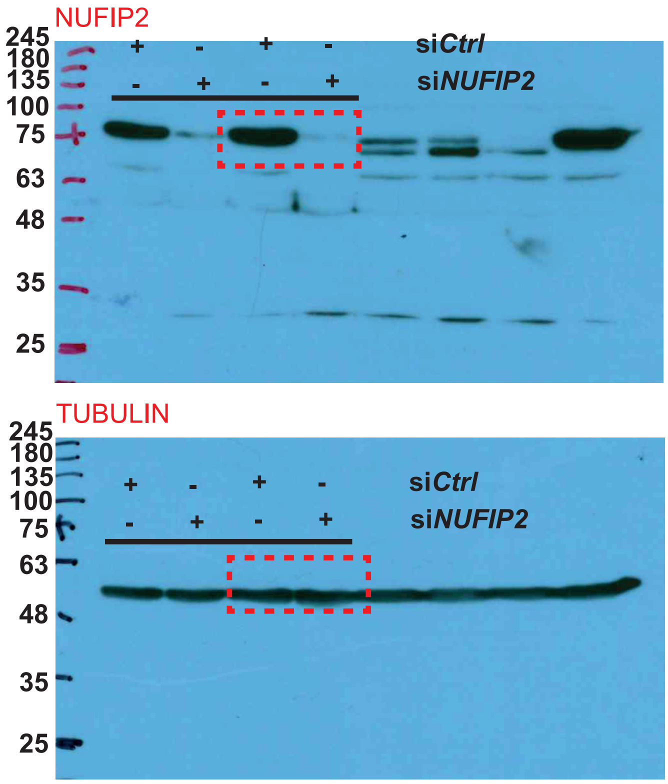

Figure 3d

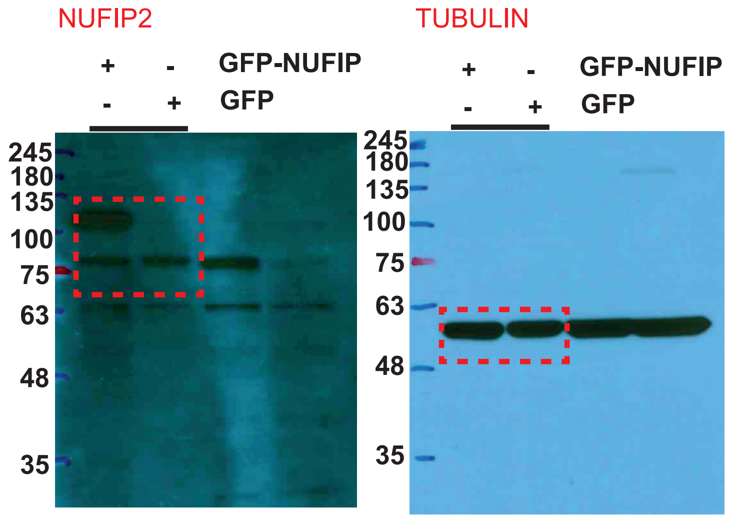

Figure 4a (Roquin-1, Roquin-2, Nufip2)

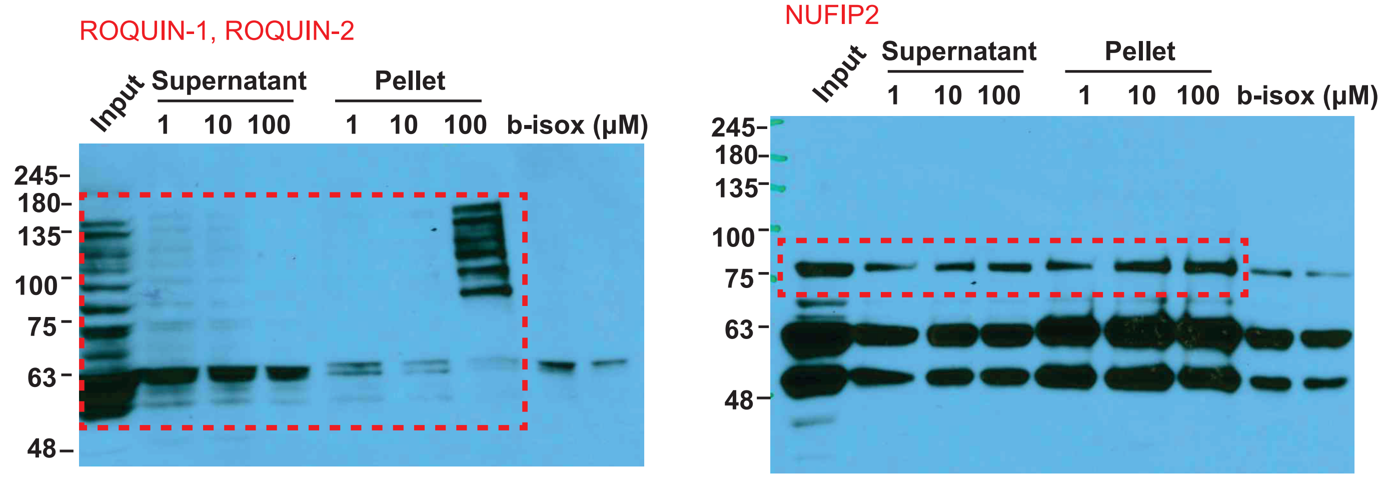

Figure 4a (Fmrp, Tubulin)

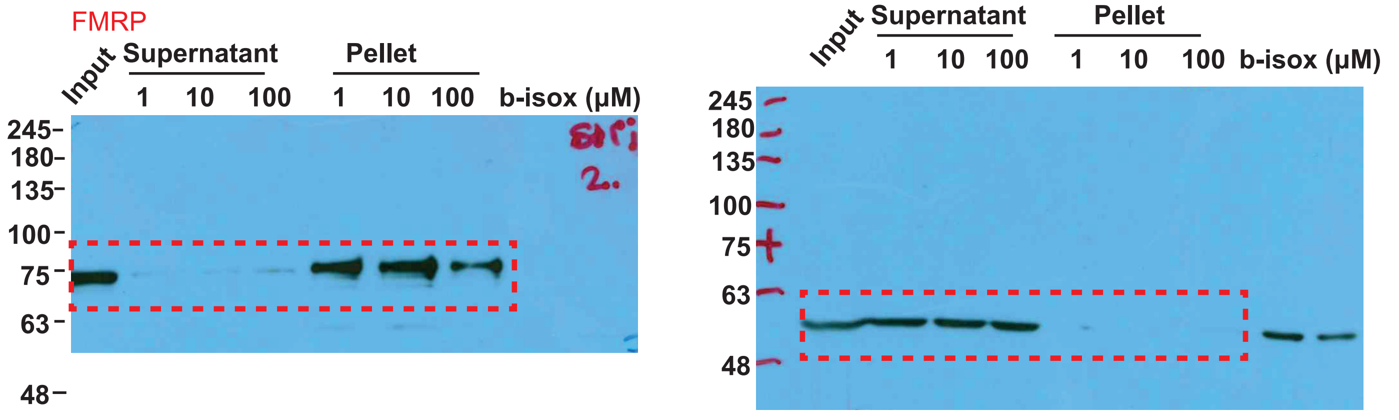

Figure 4c

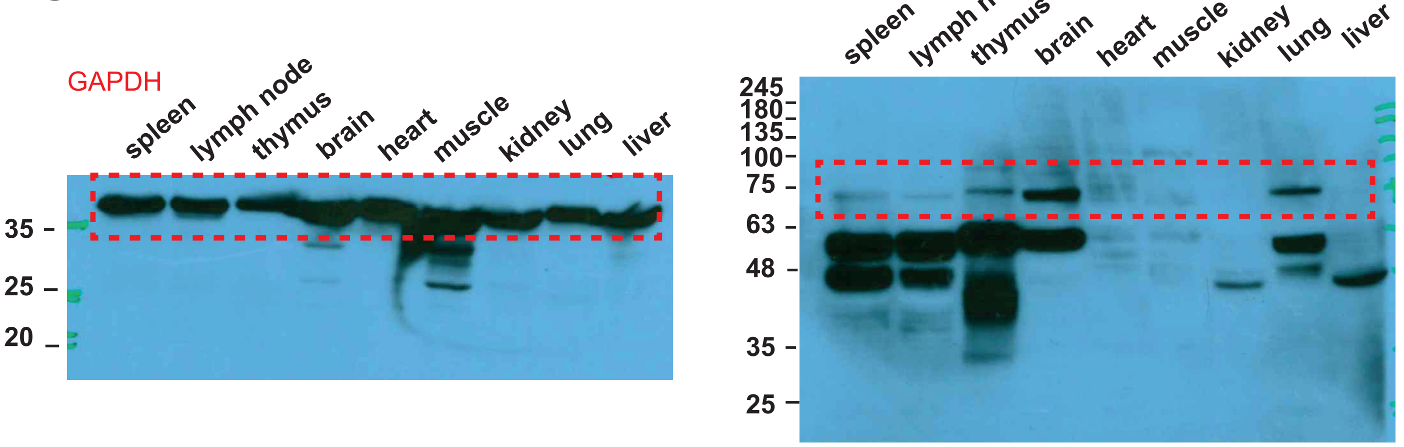

Figure 4d

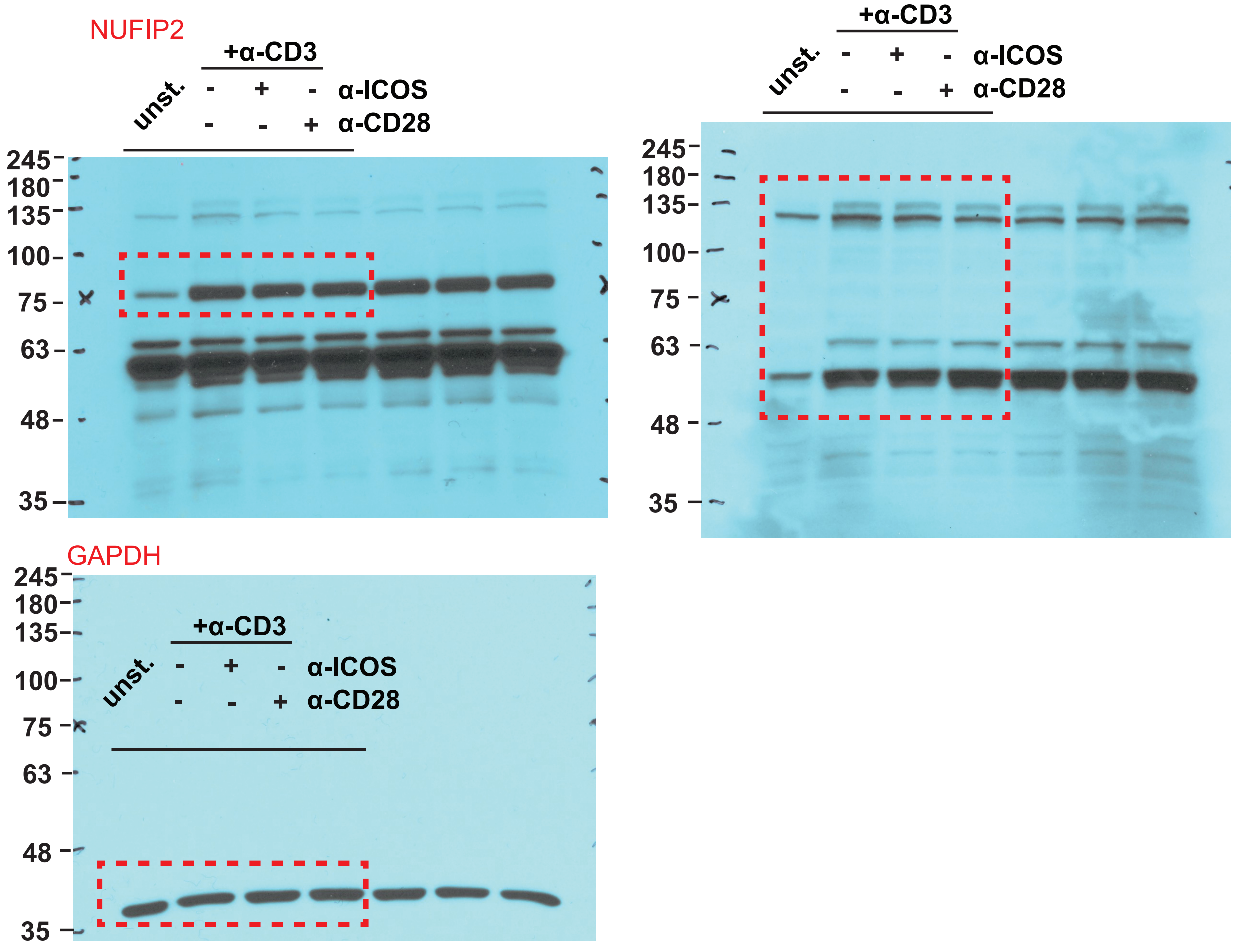

Figure 4e

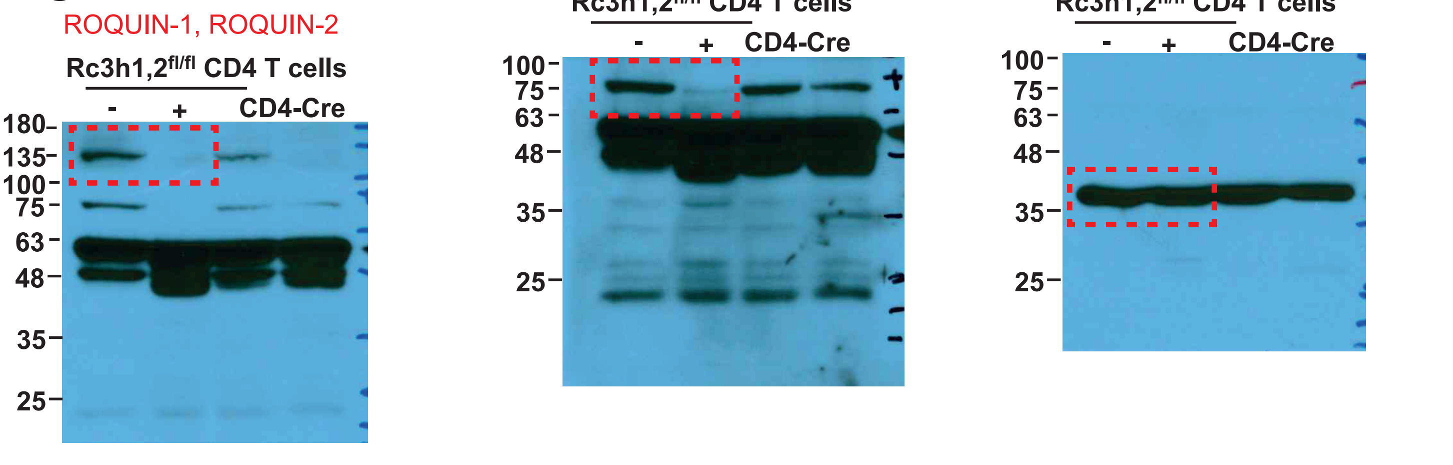

Figure 4f (Roquin-1, Roquin-2, Tubulin)

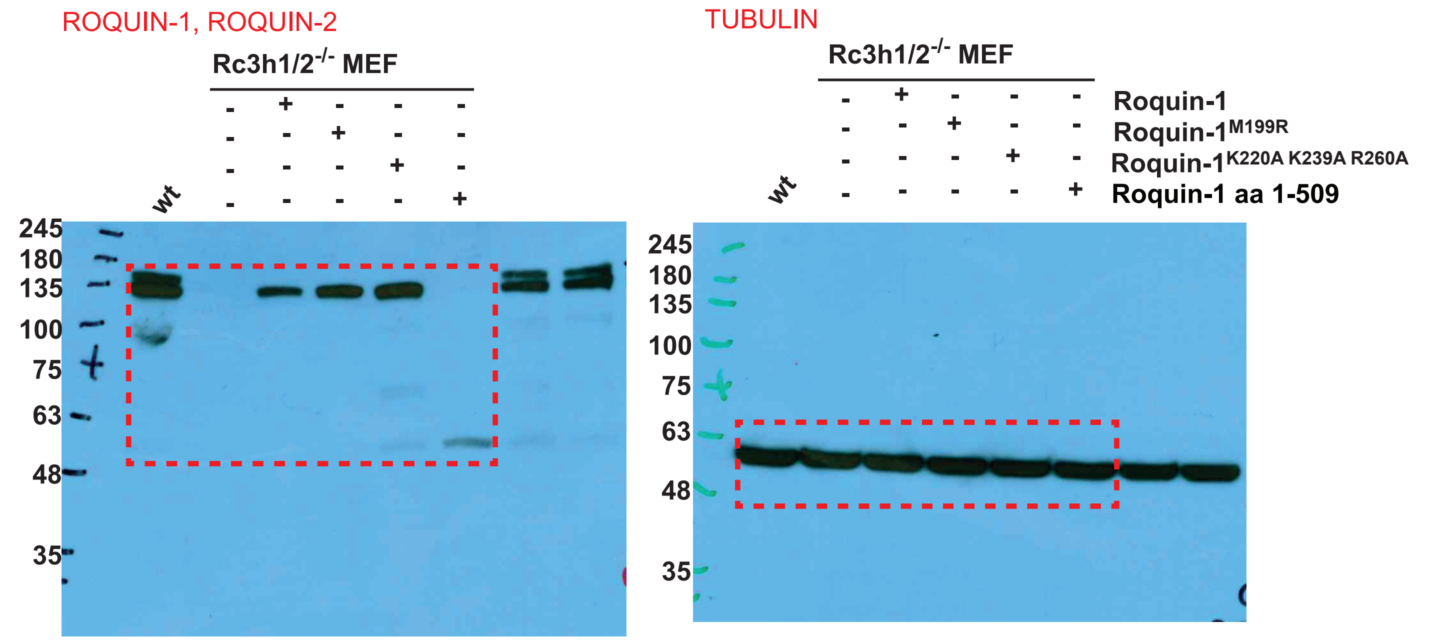

Figure 4f (Nufip2, Tubulin)

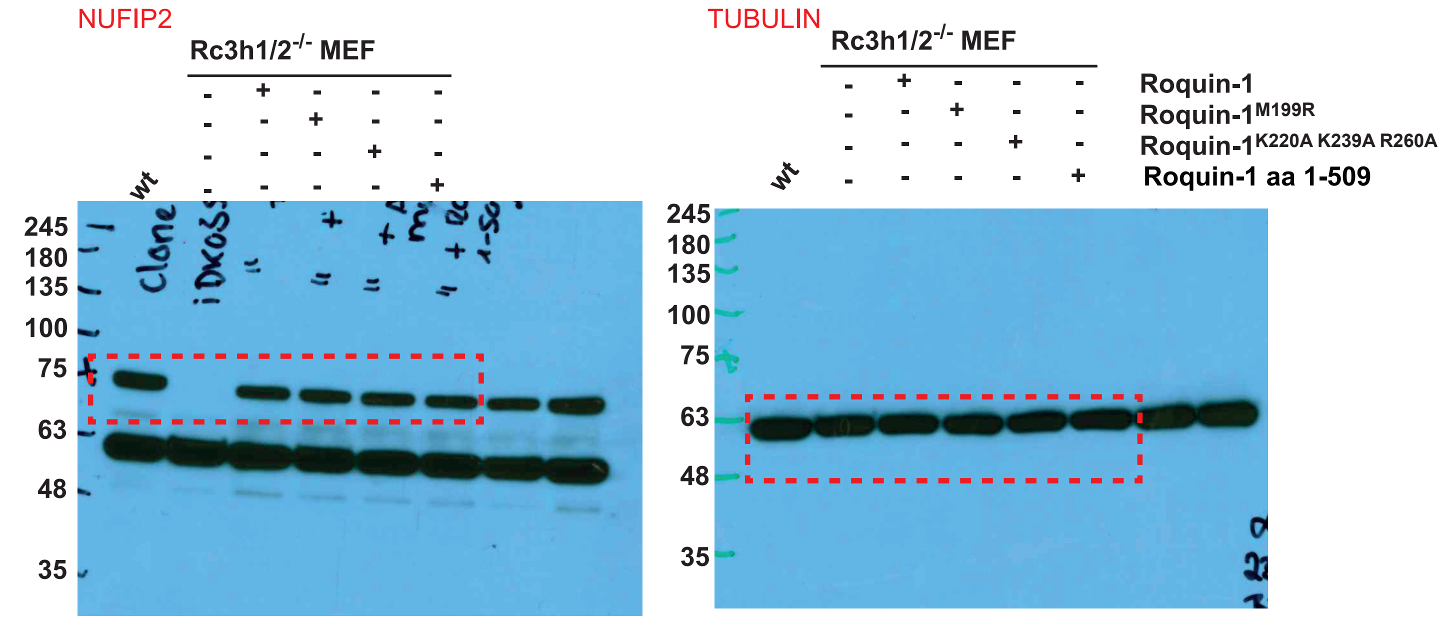

Figure 5a

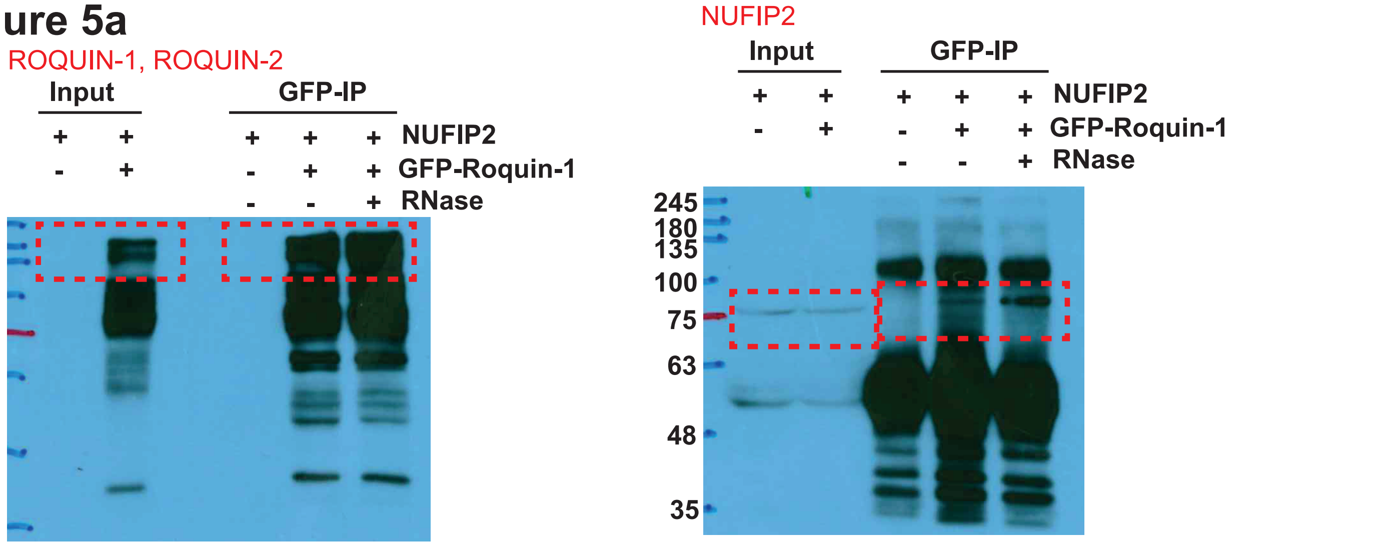

Figure 5b

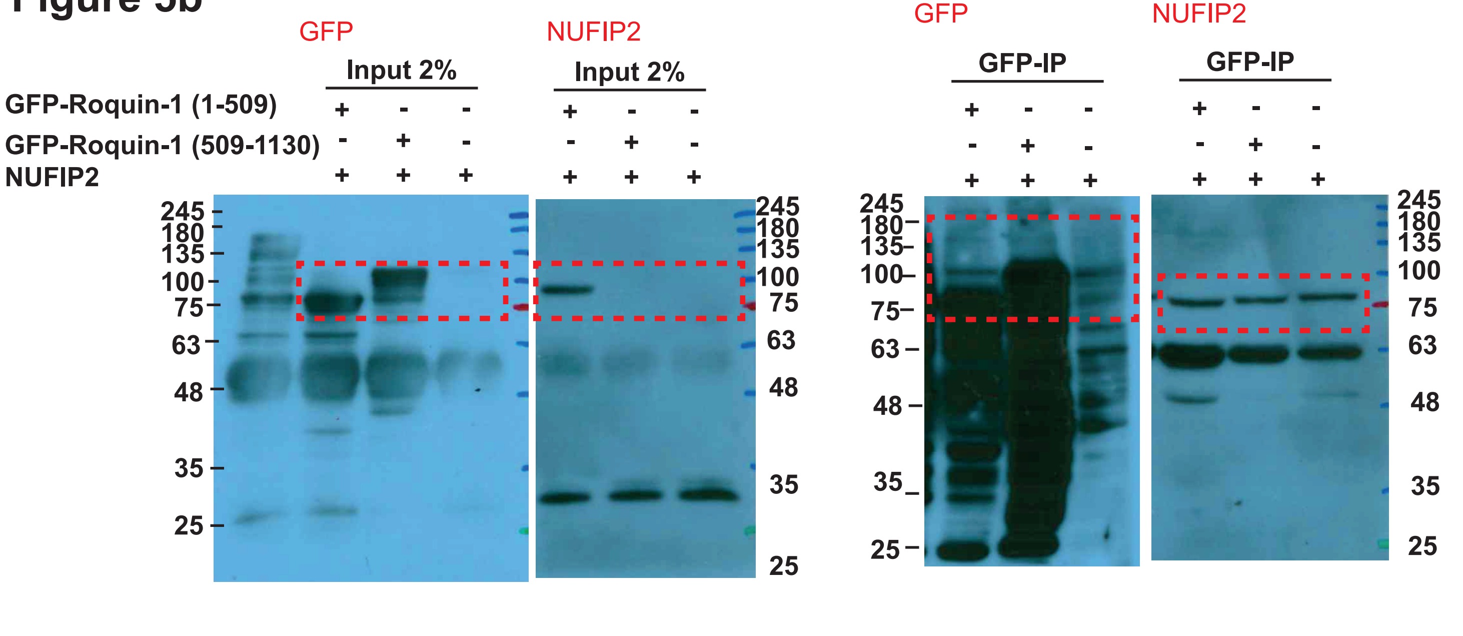

Figure 5c

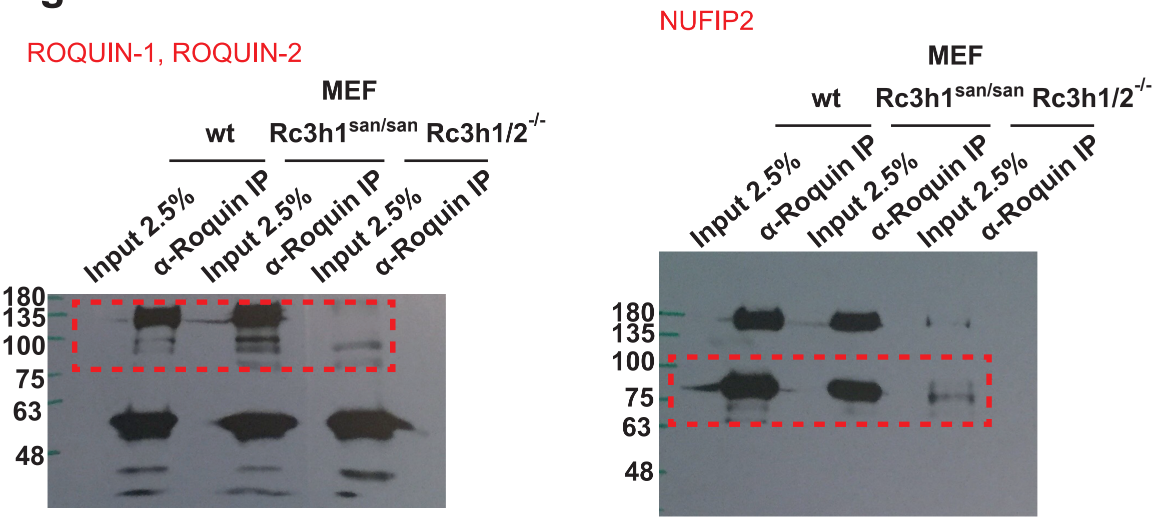

Figure 5d

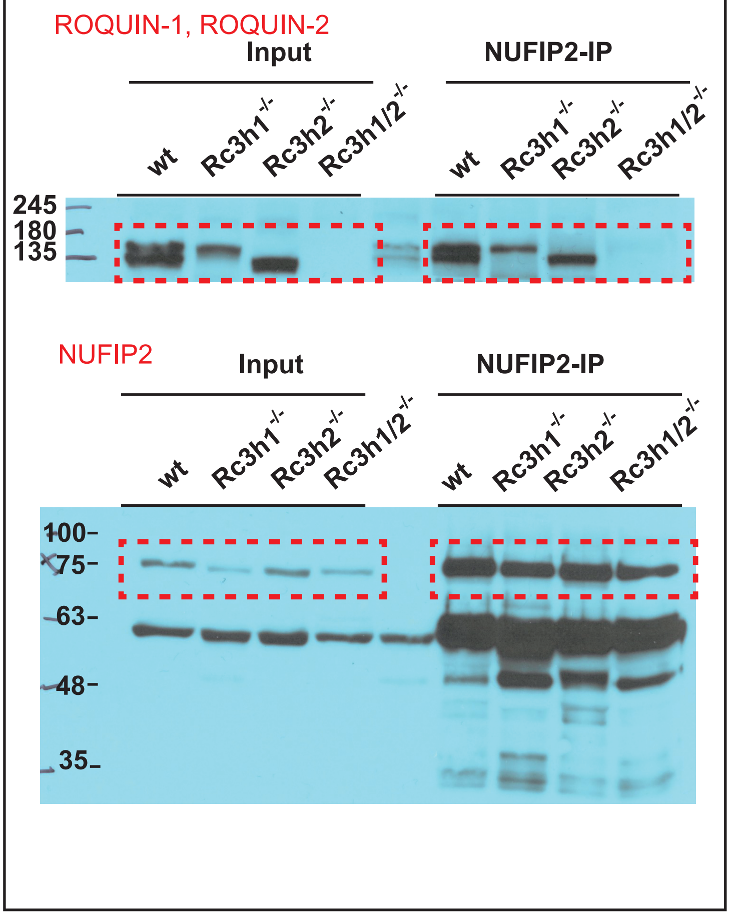

Figure 5e

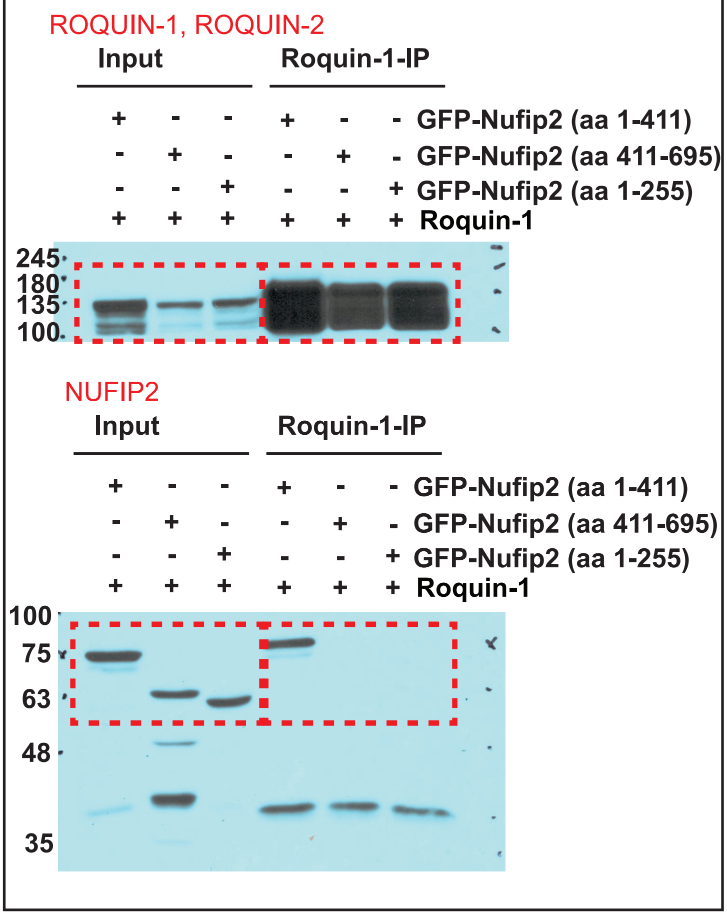

Figure 6c

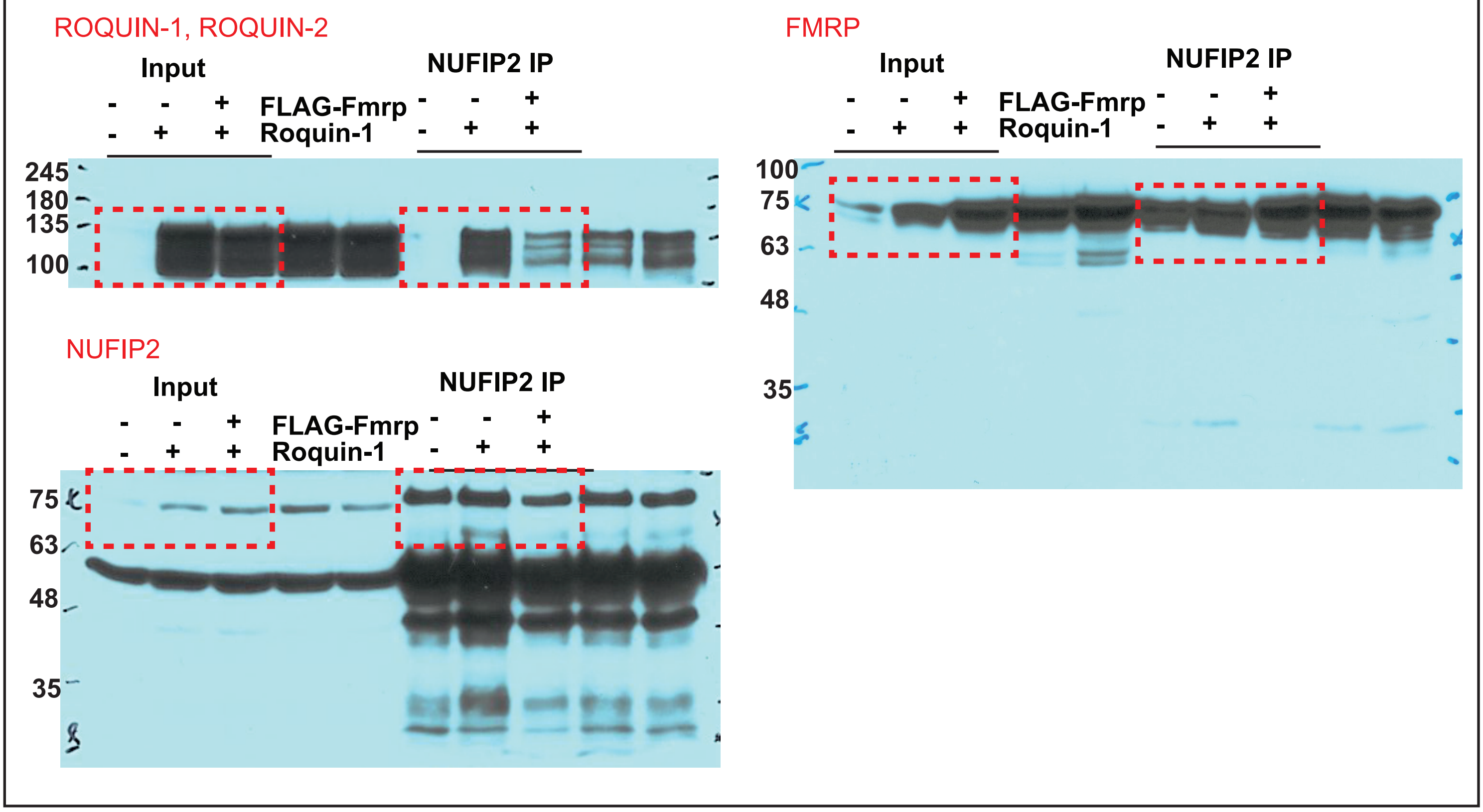

Supplementary Figure 8: Uncropped pictures of Immunoblots from main figures. Panels show original pictures of the immunoblots used to generate Figures 1a, 3c-d, 4a, c-f and 5a-c, as indicated.
