# Supplementary material for: Binding of NUFIP2 to Roquin promotes recognition and regulation of ICOS mRNA
Source: Nat Commun. 2018 Jan 19;9:299. doi: 10.1038/s41467-017-02582-1 (PMC5775257; doi:10.1038/s41467-017-02582-1)
Supplement: Supplementary file 3 — Description of Additional Supplementary Files [file 41467_2017_2582_MOESM3_ESM.pdf]

Description of Additional Supplementary Files

File Name: Supplementary Data 1

Description: Targeted siRNA screen of RNA-binding proteins. The table shows the gene ID, gene name and the experimentally determined average Z-factor for all RNA-binding proteins that were screened in their effect on Roquin-1-mediated post-transcriptional regulation of ICOS. The asterisk indicates the genes that were chosen for validation experiments by deconvolution of siRNA pools.
